# Association between serum prolactin levels and insulin resistance in non-diabetic men

**DOI:** 10.1371/journal.pone.0175204

**Published:** 2017-04-06

**Authors:** Makoto Daimon, Aya Kamba, Hiroshi Murakami, Satoru Mizushiri, Sho Osonoi, Masato Yamaichi, Kota Matsuki, Eri Sato, Jutaro Tanabe, Shinobu Takayasu, Yuki Matsuhashi, Miyuki Yanagimachi, Ken Terui, Kazunori Kageyama, Itoyo Tokuda, Ippei Takahashi, Shigeyuki Nakaji

**Affiliations:** 1 Department of Endocrinology and Metabolism, Hirosaki University Graduate School of Medicine, Hirosaki, Aomori, Japan; 2 Department of Social Medicine, Hirosaki University Graduate School of Medicine, Hirosaki, Aomori, Japan; Jichi Medical University, JAPAN

## Abstract

Prolactin (PRL) has roles in various physiological functions. Although experimental studies showed that PRL has both beneficial and adverse effects on type 2 diabetes mellitus, clinical findings in subjects with hyperprolactinemia indicate adverse effects on glucose metabolism. However, effects of PRL within the physiological range in human are controversial. A population-based study of 370 Japanese men enrolled in the 2014 Iwaki study (aged 52.0 ± 14.8 years). In this cross-sectional study, associations between serum PRL levels and homeostatic model assessment (HOMA) indices representing glucose metabolism in a physiological setting were examined using multivariable regression analysis. Although univariate linear regression analyses showed significant associations between serum PRL levels and HOMA indices, adjustment with multiple factors made the association with HOMA-ß (insulin secretion) insignificant, while those with HOMA-R (insulin resistance) remained significant (ß = 0.084, p = 0.035). Non-linear regression analyses showed a regression curve with a peak at serum PRL level, 12.4 ng/mL and a positive association of serum PRL level with HOMA-R below the peak (ß = 0.119, p = 0.004). Higher serum PRL levels within the physiological range seem to be associated with insulin resistance in men.

## Introduction

Prolactin (PRL) is a pituitary hormone known to control the initiation and maintenance of lactation [1, 2]. However, as the PRL receptor is expressed in various tissues and cells such as endometrium, the prostate, pancreatic islets, and adipocytes, PRL is also involved in various other physiological functions including metabolism [1, 3, 4]. Experimental studies indicated that PRL has effects on food intake, body weight gain, and insulin resistance via inhibiting adiponectin and IL-6 production in adipose tissue[1, 5–7], which may lead to type 2 diabetes mellitus. On the other hand, experimental studies also showed that PRL has effects on growth of pancreatic ß-cells and reduces threshold for glucose-stimulated insulin secretion [8–10], which indicate that PRL has a protective effect against type 2 diabetes mellitus. These findings suggest that in humans, the effects that PRL induces may be complex or may vary depending on different conditions.

Human studies with high serum PRL levels caused by antipsychotic drugs or prolactinoma suggested that increased levels of PRL may have adverse metabolic effects leading to type 2 diabetes [11, 12]. The findings that lowering serum PRL levels in patients with prolactinoma was accompanied by improved glucose metabolism further support its adverse effects on metabolism [11–14]. However, studies on serum PRL levels within the physiological range showed conflicting results. Some studies found a positive association between serum PRL levels and metabolic parameters such as incident hypertension [15], waist circumference [16], aortic stiffness [17], and mortality [18], as seen in individuals with pathologically high serum PRL levels. Furthermore, bromocriptine, a dopamine agonist that is well known to suppress serum PRL levels, has been shown to effectively improve insulin sensitivity, and has been approved for the treatment for type 2 diabetes mellitus in the United Sates [19]. In contrast, other studies have shown an inverse association between serum PRL levels and metabolic parameters such as cardiovascular events [20], cardiac remodeling [21], diabetes [22,23], metabolic syndrome [22,24], homeostasis model assessment (HOMA) for insulin resistance [25], and adverse lipid profiles [26]. Therefore, the association between serum PRL levels and metabolism in subjects with serum PRL levels within the physiological range requires further evaluation.

As serum PRL levels are regulated differently between genders [1], any association between serum PRL levels and any other factors should be evaluated separately for each gender. However, most previous studies have been conducted in either individual gender alone [15–18, 20, 24–26]. Some studies with subjects stratified based on gender did show gender-specific association between serum PRL levels and glucose-induced insulin release [27], cardiac remodeling [21], and metabolic syndrome [22].

Since estrogen surely have some influence both on serum PRL levels and glucose metabolism, menopausal status and ovary cycle phase appear to be important. However, we did not monitor menopausal status and ovary cycle phase. Therefore, we here focused on examining the association between serum PRL levels within the physiological range and glucose metabolism in a general population in men.

## Subjects and methods

### Subjects

The Iwaki study is a health promotion study of Japanese people over 20 years of age with the aim of preventing lifestyle-related diseases and prolonging lifespan. It is held annually in the Iwaki region of Hirosaki in Aomori Prefecture located in northern Japan [28]. In 2014, 441 men were enrolled in the Iwaki study. Of these, the followings were excluded from the current study: 9 with serum PRL levels over 25 ng/mL, 45 with diabetes, and 17 with a fasting blood glucose level below 63 mg/dL, to better evaluate HOMA indices. Diabetes was defined according to 2010 Japan Diabetes Society criteria (fasting blood glucose level ≥ 126 mg/dL) [29]. In subjects whose fasting blood glucose level was not measured, diabetes was defined as HbA1c ≥ 6.5%. Those on medication for diabetes were also defined as having diabetes. After exclusions, 370 individuals aged 52.0 ± 14.8 years were included in the study.

This study was approved by the Ethics Committee of Hirosaki University School of Medicine and was conducted in accordance with the principles contained in the Declaration of Helsinki. Written informed consent was obtained from all participants.

### Clinical characteristics

Blood samples were collected in the morning from peripheral veins of participants under fasting conditions in a supine position for 5 min. after 10 min. rest in a sitting position. Serum PRL levels were determined using chemiluminescence-immunoassay in a commercial laboratory (LSI Medience Corp., Tokyo, Japan) (details of the assays are in S1 File). The following clinical characteristics were measured: height, body weight, body mass index (BMI), fasting serum glucose, fasting serum insulin level, glycated hemoglobin (HbA1c), systolic blood pressure, diastolic blood pressure, total serum levels of total cholesterol, triglyceride, high-density lipoprotein-cholesterol, uric acid, urea nitrogen, creatinine, adiponectin, and leptin. HbA1c (%) is expressed as the National Glycohemoglobin Standardization Program value. Insulin resistance and secretion were assessed by homeostasis model assessment using fasting blood glucose and insulin levels (HOMA-R and HOMA-ß, respectively). Hypertension was defined as blood pressure ≥140/90 mmHg or taking treatment for hypertension (n = 171). Hyperlipidemia was defined as total cholesterol ≥220 mg/dL, triglyceride ≥150 mg/dL or taking treatment for hyperlipidemia. (n = 156). Alcohol intake (current or non-drinker) and smoking habits (never, past or current) were determined from questionnaires.

### Statistical methods

Clinical characteristics are given as means ± SD. The statistical significance of differences in characteristics values between two groups (parametric) and a case-control association between groups (nonparametric) were assessed by analysis of variance and χ^2^ tests, respectively. Correlations between serum PRL levels and clinical characteristics, including HOMA indices, were assessed by linear regression analyses. In addition, non-linear regression analyses with curve fitting with the Gaussian model were used to assess correlation between serum PRL levels and HOMA indices. For statistical analyses, HOMA indices, and serum levels of PRL, adiponectin, and leptin were log-transformed (log10) to approximate a normal distribution. A p<0.05 was considered statistically significant. Non-linear regression analyses were performed using JMP software version 12.0.1(SAS Institute Inc., Cary, NC). All other analyses were done using StatView software (ver. 5.0; SAS Institute Inc., Cary, NC).

## Results

### Clinical characteristics of study subjects

The clinical characteristics of subjects are shown in Table 1. Mean ages were 52.0 ± 14.8. Serum PRL levels were s7.06 ± 3.37.

**Table 1:** Characteristics of the study subjects.

Characteristics	
**Number**	**370**
**Age (yr)**	**52.0±14.8**
**Height (cm)**	**168.6±6.6**
**Body weight (kg)**	**66.7±9.7**
**Body mass index (kg/m2)**	**23.4±3.0**
**Fat (%)**	**18.7±5.6**
**Prolactin:PRL (ng/ml)**	**7.06±3.37**
**HbA1c (%)**	**5.63±0.30**
**Fasting serum glucose (mg/dl)**	**80.9±9.2**
**Fasting serum insulin:IRI (μU/ml)**	**4.26±2.28**
**HOMA-R**	**0.84±0.50**
**HOMA-ß (%)**	**116.2±145.9**
**Systolic blood pressure (mmHg)**	**132.7±18.0**
**Diastolic blood pressure (mmHg)**	**80.6±11.2**
**Total cholesterol (mg/dl)**	**197.8±33.5**
**Triglyceride (mg/dl)**	**125.5±108.1**
**HDL cholesterol (mg/dl)**	**59.5±16.8**
**Serum uric acid (mg/dl)**	**5.94±1.23**
**Serum urea nitrogen (mg/dl)**	**15.2±4.3**
**Serum creatinin (mg/dl)**	**0.82±0.15**
**Adiponectin (mg/dl)**	**8.24±3.69**
**leptin (ng/ml)**	**3.58±2.21**
**Hypertension: n (%)**	**171 (46.2)**
**Hyperlipidemia: n (%)**	**156 (42.2)**
**Drinking alcohol: n (%)**	**256 (69.2)**
**Smoking (Never/ Past/ Current):n**	**128/127/114**

HbA1c: glycated hemoglobin; HDL: high-density lipoprotein.

### Correlations between serum PRL levels and HOMA indices (insulin resistance and secretion)

Correlations between clinical characteristics and HOMA-R (representing insulin resistance) are shown in S1 Table. Serum PRL levels significantly correlated with HOMA-R, even after adjustments for multiple factors correlated with HOMA-R in univariate correlation analyses (ß = 0.084, p = 0.035). Sensitivity analysis without HbA1c as a covariate did not change the correlation (β = 0.081, p = 0.043).

Correlations between clinical characteristics and HOMA-ß (representing insulin secretion) were also examined (S1 Table). Although univariate regression analyses revealed correlations between serum PRL levels and HOMA-ß (ß = 0.108, p = 0.037), such correlations became insignificant after adjustment for multiple factors correlated with HOMA-ß in univariate correlation analyses.

**Fig 1:**
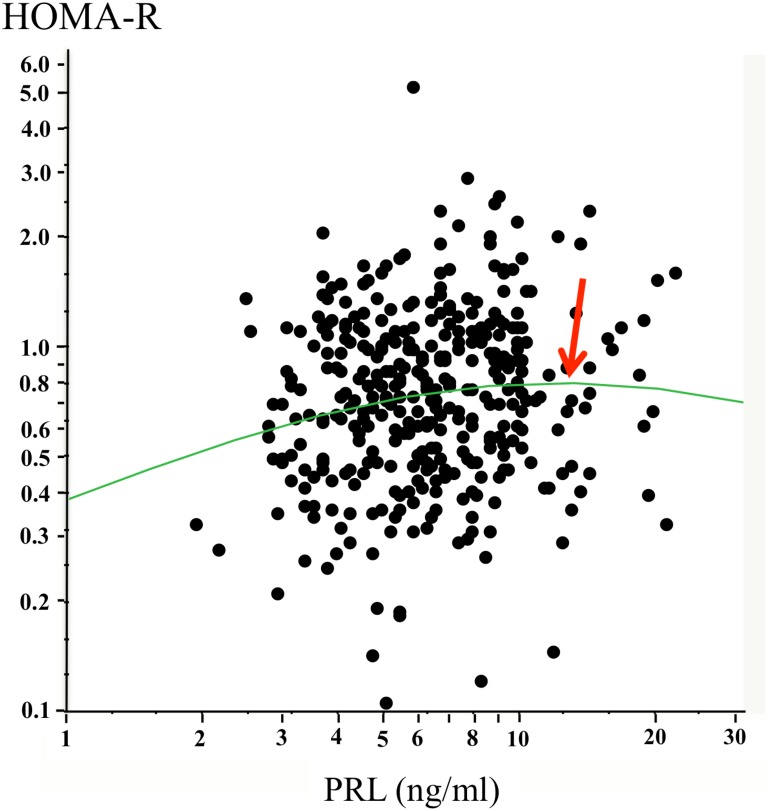
Non-linear regression analyses with curve fitting with the Gaussian model. Correlations between serum prolactin (PRL) levels and insulin resistance index assessed by homeostasis model (HOMA-R) are shown. The arrow indicates a peak on the regression curve for men at a serum PRL level 12.4 ng/mL.

As shown in Fig 1, graphs of the data points appeared to support such correlation between serum PRL levels and HOMA-R. Further, the graph indicates that the correlation may not form a straight line. Thus, we examined the correlations with non-linear regression analyses, which showed a regression curve with a peak at a serum PRL level of 12.4 ng/mL. When subjects were stratified based on serum PRL levels, p values for the positive correlation between serum PRL level and HOMA-R decreased further, even after adjustment for multiple factors in subjects whose serum PRL level was below the peak (ß = 0.119, p = 0.004), while such correlations were not observed in subjects whose serum PRL level was above the peak (Table 2). These findings were further verified by additional analyses in which subjects were divided into halves (high vs. low) in each stratified group. From these analyses, it was found that high serum PRL levels significantly correlated with HOMA-R even after adjustment for multiple factors in subjects whose serum PRL level was below the peak (ß = 0.082, p = 0.046), while such correlations were not observed in subjects whose serum PRL level was above the peak (Table 2).

**Table 2:** Correlation between serum PRL levels and HOMA-R stratified by serum PRL.

	≦12.4(n = 343)			>12.4(n = 27)		
	Simple	Age adjusted	Multiple factors	Simple	Age adjusted	Multiple factors
**Serum PRL level**	0.150*	0.151*	0.119*	**0.175**	**-0.199**	**0.263**
**PRL (High vs Low)**	0.113*	0.113*	0.082*	**0.331**	**0.333**	**0.318**

Correlation coefficients (r) are shown. p-values <0.05 obtained by regression analysis are indicated by *. Multiple factors: adjusted with age, body mass index, glycated hemoglobin, serum levels of total and HDL cholesterol, and leptin, and alcohol consumption.

## Discussion

In this cross-sectional study of a non-diabetic Japanese population, we examined the association of serum PRL levels within the physiological range with glucose metabolism in men, and found a positive association with HOMA-R with serum PRL levels below 12.4 ng/mL, which was found as a peak by non-linear regression analysis. These results indicate that higher serum PRL levels within the physiological range is associated with insulin resistance in men.

As shown in Table 1, distributions of values of HOMA indices were very limited, and, thus, the statistical power to evaluate relationship between serum PRL levels and HOMA indices appeared to be low. Therefore, we experimentally expanded the sample population by adding those with diabetes without its medication and those with serum PRL levels between 25 and 50 ng/ml (namely, 3 with serum PRL levels over 50 ng/mL, 32 on medication for diabetes, and 20 with a fasting blood glucose level below 63 mg/dL or over 140 mg/dL, to better evaluate HOMA indices, were excluded from the original sample set of 441)(n = 386). Analyses with this sample set verified the positive association between serum PRL levels and HOMA-R with serum PRL levels below a peak determined by non-linear regression analysis (S2 Table). Furthermore, although it is very arbitrary, when insulin resistance was defined as HOMA-R ≥1.6, to increase number of such subjects (n = 33) and thus increase statistical power, multiple logistic regression analyses showed that high serum PRL levels were significantly associated with insulin resistance (OR: 2.67, 95% CI: 1.01–7.06) in men with a serum PRL level below the peak (S3 Table).

Gender-specific effects of PRL have been reported in experimental and human studies. Suppression of PRL release by bromocriptine has been reported as most effective in lactating rats and less effective in males [30]. Further, in human studies of hyperprolactinemia, bromocriptine reduced body weight more effectively in men than in women [31–33]. Associations of serum PRL levels with factors related to metabolism have been reported in several human studies with subjects with normal serum PRL levels. Although most such studies were conducted in at least one of the following conditions, relatively small, selected patient populations, either gender alone, or without adjustment for confounding factors [15–18, 20, 24–26], gender-specific associations have been reported in a large population-based study, where although inverse associations between serum PRL levels and type 2 diabetes mellitus were found in both genders, a significant trend for higher serum PRL levels with decreasing number of metabolic syndrome components was found in women but not in men [22]. With regard to type 2 diabetes mellitus, the results of that study were different from ours. Although we did not directly examine association between serum PRL levels and type 2 diabetes, we evaluated association between serum PRL levels and HOMA indices, which seem to be surrogate markers for type 2 diabetes, and found positive association between serum PRL levels and HOMA-R in men. Such differences may come from differences in study population. Namely, changes resulting as secondary from diabetic conditions might substantially influence the differences in the results. Alternatively, our results appear to reflect non-diabetic conditions, and may therefore indicate mechanisms linking PRL to type 2 diabetes mellitus (insulin resistance) more precisely than the previous study.

It is possible that estrogen may be responsible for the gender-specific associations previously reported, because estrogen is believed to play a central role in the control of PRL release. However, this suggestion is mostly based on rodent data, with there being little direct human data, and, thus, endogenous estrogen may have only a modest effect on stimulation of PRL release in normal human subjects [1]. Although estrogen *per se* is known to have insulin-sensitizing properties and to have a protective effect on pancreatic ß-cells, associations between estrogen levels and diabetes are conflicting [34]. Therefore, mechanisms involved in gender-specific association do not appear to be explained by estrogen levels alone; further studies are needed to elucidate such mechanisms.

Pituitary PRL release is regulated by the dopaminergic neuronal system via dopamine receptor type 2 (D2R), the activation of which inhibits PRL release as well as regulating many other functions such as cognition, motivation, fitness, appetite, and energy homeostasis [1, 27, 35]. An experimental study that examined genetic disruption of D2R resulted in glucose intolerance with impaired insulin secretion [36]. Administration of antipsychotic drugs, which block dopamine receptors, can result in weight gain and impaired glucose tolerance [27, 35]. Conversely, bromocriptine improves glycemic control in patients with type 2 diabetes mellitus, and, is currently used as a drug for the treatment of type 2 diabetes [19]. Serum PRL levels can be a reflection of dopaminergic tone, therefore, the observed association between serum PRL levels and HOMA-R may be merely a reflection of the association between dopaminergic tone and HOMA-R. This possibility is warranted for evaluation in the future.

Although PRL acts as a circulating hormone in most animals, in humans, PRL is produced by multiple tissues (breast, skin, prostate, and adipose tissue) and cells (decidua, miometrium, lymhocytes, and adipocytes), and can therefore act as a cytokine [1]. The production of PRL in human adipose tissue is of particular interest as adipose tissue plays a pivotal role in metabolism. PRL seems to be released proportionally to the quantity of fat mass in obese women, and weight loss in such subjects resulted in a decrease in 24-h PRL release [37, 38]. Further, macrophages derived from adipose tissue were shown to synthesize PRL in response to inflammation and high glucose concentrations [39]. Therefore, obesity and/or higher glucose levels (or glycemic levels) seem to influence the observed association between serum PRL levels and HOMA-R. However, adjustments with factors related to obesity and glycemic control used for the analyses appear to indicate the observed association as being independent and, thus, fundamental.

A strength of the current study is that in addition to ordinary linear regression analyses, non-linear regression analyses were applied. This is the first time, to the best of the authors’ knowledge, that such analyses have been reported. Such results appear to be determined only with the kinds of analyses conducted in this study. Further, statistical adjustments were made for multiple factors that could confound the results, and a relatively large population-based/general sample of individuals was used. Additionally, subjects on medication for diabetes were excluded, as these drugs affect glycemic parameters including HOMA indices. Therefore, the results obtained appear to reflect the relationship between serum PRL levels and HOMA indices precisely. Furthermore, the study population appears to represent general Japanese population, at least, in regard with prevalence of life-style related disease. Prevalence of hypertension and hyperlipidemia were 46.2% and 41.4%, respectively. The prevalence of hypertension is not so different from the 2010 national values aged 30 to 69 years reported by the Japanese government (50.8%) [40]. There are no reported national values for hyperlipidemia prevalence (using the same definition as this study), but our observed prevalence is similar to that reported in other areas of Japan [25]. Further, although no diabetic subjects were included in the study sample, the prevalence of diabetes in our original sample was 11.7%, which are similar to Japanese national values [40].

This study had some limitations. The subjects were participants in a health promotion study rather than an ordinary health check-up study, and may not therefore accurately represent the general population. Indeed, the number of women participating was much higher than that of men participating. Therefore, they may be more invested in keeping themselves healthy compared with the general population, although the prevalence of life-style-related diseases do not appear to be different form national values in Japan. As described previously, estrogen surely have some influence both on serum PRL levels and glucose metabolism, and, thus, menopausal status and ovary cycle phase appear to be important. However, we did not monitor menopausal status nor ovary cycle phase. Therefore, we could not examine any association between serum PRL levels and HOMA indices in women, and, thus, this issue in women remained to be elucidated in the future. Further, because PRL is expressed in extra-pituitary organs/tissues and acts as a cytokine in humans, local expression and/or levels of PRL may have influences on the results. However, as these levels cannot be determined readily, such evaluation was not possible in our study. Finally, as our study was cross-sectional and not a cohort study, we could not assess the risk of serum PRL levels on the future incidence of diabetes.

In conclusion, associations between serum PRL levels within the physiological range and HOMA-R were found to be positive for men. These findings indicate that higher serum PRL levels within the physiological range seem to be associated with insulin resistance in men in this non-diabetic Japanese population. Further studies are required to determine whether such findings are applicable to other ethnicities and whether the associations can be exploited to predict the risk for the incidence of type 2 diabetes mellitus.

## Supporting information

S1 File Analytical performance (PRL). (DOCX)

S1 Table Correlation between serum PRL levels and HOMA indices in men. (DOCX)

S2 Table Correlation between serum PRL levels and HOMA-R in men stratified based on the serum PRL levels. (DOCX)

S3 Table Correlation between serum PRL levels and insulin resistance in men. (DOCX)
