# Supplementary material for: Association between serum prolactin levels and insulin resistance in non-diabetic men
Source: PLoS One. 2017 Apr 6;12(4):e0175204. doi: 10.1371/journal.pone.0175204 (PMC5383244; doi:10.1371/journal.pone.0175204)
Supplement: S1 File — (DOCX) [file pone.0175204.s001.docx]

**Analytical Performance**

**PRL**

PRL is measured by Chemiluminescent Microparticle Immunoassay (CMIA) using The ARCHITECT Prolactin assay kit on the Architect i2000 immunoassay analyzer (Abbott Japan Co., Chiba, Japan).

The assay detection limit is 0.6 ng/mL.

Measurement range: 0.6~200 ng/ml

Upper limit with automated dilution: 2000 ng/ml

Intra-assay precision: 2.3～3.8%, inter-assay precision: 3.3～4.7%.
