# Supplementary material for: Association between serum prolactin levels and insulin resistance in non-diabetic men
Source: PLoS One. 2017 Apr 6;12(4):e0175204. doi: 10.1371/journal.pone.0175204 (PMC5383244; doi:10.1371/journal.pone.0175204)
Supplement: S1 Table — (DOCX) [file pone.0175204.s002.docx]

**Supplemental Table 1. Correlation between serum PRL levels and HOMA indices in men**

|  | | | | | |  |
| --- | --- | --- | --- | --- | --- | --- |
|  | HOMA-R | |  | HOMA-** β** | | |
| Characteristics | Univariate | Multivariate |  | Univariate | Multivariate | |
| r^2^ of the test | - | 0.480 |  | - | 0.368 | |
| Serum PRL (ng/ml) | 0.133* | 0.084* |  | 0.108* | -0.002 | |
| Age (yr) | -0.026 | 0.029 |  | -0.455* | -0.259* | |
| Height (cm) | -0.016 | - |  | 0.187* | - | |
| Body weight (kg) | 0.479* | - |  | 0.373* | 0.131* | |
| Body mass index (kg/m^2^) | 0.559* | 0.321* |  | 0.311* | - | |
| Fat (%) | 0.546* |  |  | 0.244* | - | |
| HbA1c (%) | 0.233* | 0.118* |  | -0.252* | -0.185* | |
| Systolic blood pressure (mmHg) | 0.170* | - |  | -0.112* | -0.023 | |
| Diastolic blood pressure (mmHg) | 0.160* | 0.059 |  | -0.033 | - | |
| Total cholesterol (mg/dl) | 0.232* | 0.118* |  | 0.076 | 0.074 | |
| Triglyceride (mg/dl) | 0.202* |  |  | 0.204* | - | |
| HDL cholesterol (mg/dl) | -0.263* | -0.136* |  | -0.235* | -0.153* | |
| Serum uric acid (mg/dl) | 0.129* | -0.070 |  | 0.137* | 0.017 | |
| Serum urea nitrogen (mg/dl) | 0.038 | - |  | -0.276* | -0.091 | |
| Serum creatinine (mg/dl) | 0.159* | 0.081 |  | 0.069 | - | |
| Adiponectin (10 x log mg/dl) | -0.277* | -0.041 |  | -0.289* | -0.070 | |
| Leptin (ng/ml) | 0.538* | 0.264* |  | 0.278* | 0.179* | |
| Hypertension: n (%) | 0.210* | - |  | -0.113* | - | |
| Hyperlipidemia: n (%) | 0.275* | - |  | 0.183* | - | |
| Drinking alcohol: n (%) | -0.187* | -0.164* |  | -0.117* | -0.089* | |
| Smoking (Never/ Past/ Current) | -0.114* | - |  | 0.067 | 0.009 | |
|  | | | | | | |

HbA1c: glycated hemoglobin; HDL: high-density lipoprotein. Correlation coefficients are shown. p-values <0.05 obtained by regression analysis are indicated by *. Characteristics indicated by - are those not included in multiple regression analysis.
