# Supplementary material for: Association between serum prolactin levels and insulin resistance in non-diabetic men
Source: PLoS One. 2017 Apr 6;12(4):e0175204. doi: 10.1371/journal.pone.0175204 (PMC5383244; doi:10.1371/journal.pone.0175204)
Supplement: S2 Table — (DOCX) [file pone.0175204.s003.docx]

**Supplemental Table 2. Correlation between serum PRL levels and HOMA-R in men stratified based on the serum PRL levels**

|  | | | | | | | | |
| --- | --- | --- | --- | --- | --- | --- | --- | --- |
|  | Serum PRL levels | | | | | | | |
|  | <9.8(n=322) | | |  | | >9.8(n=64) | | |
|  | Simple | Age adjusted | Multiple factors |  | | Simple | Age adjusted | Multiple factors |
| Serum PRL level | 0.179* | 0.181* | 0.163* |  | | -0.08 | -0.082 | 0.0005 |
|  | | | | | | | | |
| PRL (High vs Low) | 0.169* | 0.171* | 0.131* |  | -0.064 | | -0.079 | -0.022 |
|  | | | | | | | | |

Correlation coefficients are shown. p-values <0.05 obtained by regression analysis are indicated by *. Multiple factors: adjusted with age, body mass index, glycated hemoglobin, serum levels of creatinine, adiponectin, and leptin, and alcohol consumption.
