# Supplementary material for: Association between serum prolactin levels and insulin resistance in non-diabetic men
Source: PLoS One. 2017 Apr 6;12(4):e0175204. doi: 10.1371/journal.pone.0175204 (PMC5383244; doi:10.1371/journal.pone.0175204)
Supplement: S3 Table — (DOCX) [file pone.0175204.s004.docx]

**Supplemental Table 3. Correlation between serum PRL levels and insulin resistance in men**

|  | **Serum PRL levels (<9.8)** | | |  |
| --- | --- | --- | --- | --- |
|  | **OR** | **95%CI** | **p** |  |
| **Serum PRL levels**  **(per 0.1 logPRL)** | **1.79** | **1.23-2.62** | **0.003** |  |
| **PRL (High vs Low)** | **2.67** | **1.01-7.06** | **0.047** |  |
|  | | | | |

Insulin resistance was defied as HOMA-R >1.6. Adjusted with age, body mass index, glycated hemoglobin, serum levels of creatinine, adiponectin, and leptin, and alcohol consumption.
